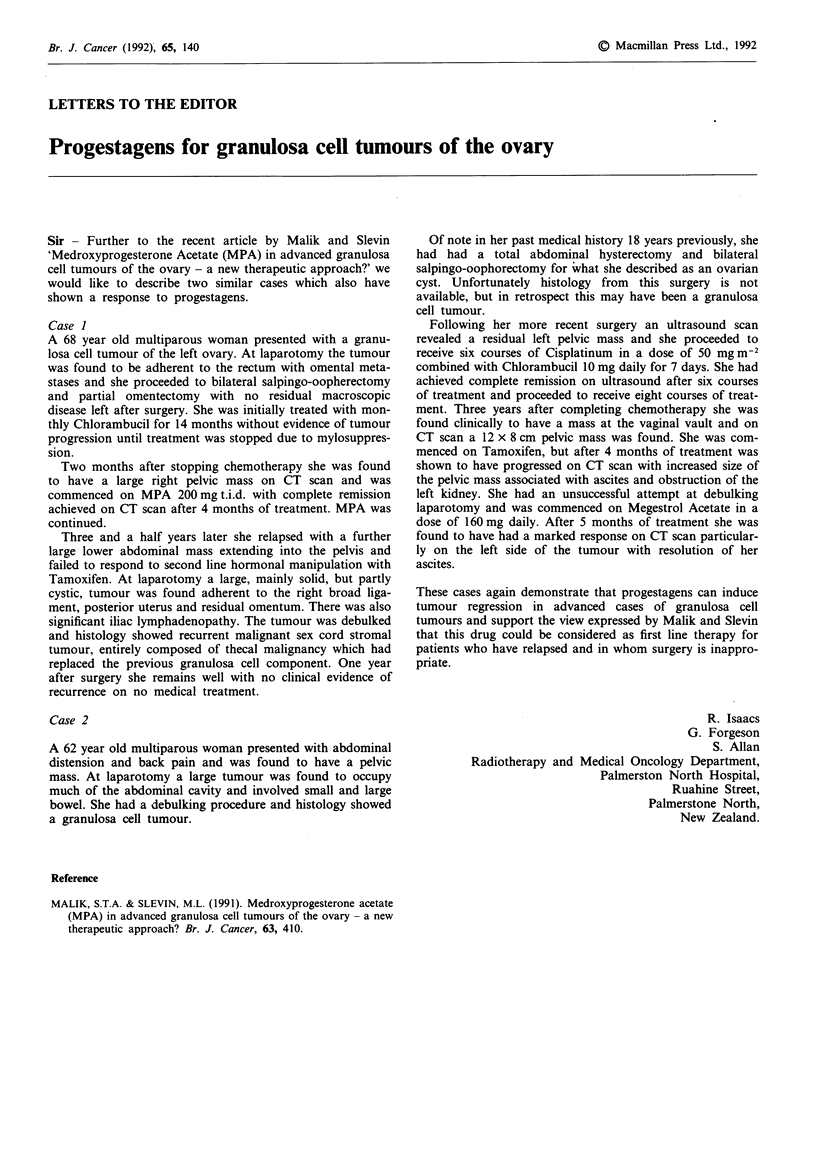# Progestagens for granulosa cell tumours of the ovary.

**DOI:** 10.1038/bjc.1992.28

**Published:** 1992-01

**Authors:** R. Isaacs, G. Forgeson, S. Allan


 © Macmillan Press Ltd., 1992

### LETTERS TO THE EDITOR

### Progestagens for granulosa cell tumours of the ovary

**Sir** – Further to the recent article by Malik and Slevin 'Medroxyprogesterone Acetate (MPA) in advanced granulosa cell tumours of the ovary – a new therapeutic approach?' we would like to describe two similar cases which also have shown a response to progestagens.

*Case 1*

A 68 year old multiparous woman presented with a granulosa cell tumour of the left ovary. At laparotomy the tumour was found to be adherent to the rectum with omental metastases and she proceeded to bilateral salpingo-oopherectomy and partial omentectomy with no residual macroscopic disease left after surgery. She was initially treated with monthly Chlorambucil for 14 months without evidence of tumour progression until treatment was stopped due to mylosuppression.

Two months after stopping chemotherapy she was found to have a large right pelvic mass on CT scan and was commenced on MPA 200 mg t.i.d. with complete remission achieved on CT scan after 4 months of treatment. MPA was continued.

Three and a half years later she relapsed with a further large lower abdominal mass extending into the pelvis and failed to respond to second line hormonal manipulation with Tamoxifen. At laparotomy a large, mainly solid, but partly cystic, tumour was found adherent to the right broad ligament, posterior uterus and residual omentum. There was also significant iliac lymphadenopathy. The tumour was debulked and histology showed recurrent malignant sex cord stromal tumour, entirely composed of thecal malignancy which had replaced the previous granulosa cell component. One year after surgery she remains well with no clinical evidence of recurrence on no medical treatment.

*Case 2*

A 62 year old multiparous woman presented with abdominal distension and back pain and was found to have a pelvic mass. At laparotomy a large tumour was found to occupy much of the abdominal cavity and involved small and large bowel. She had a debulking procedure and histology showed a granulosa cell tumour.

Of note in her past medical history 18 years previously, she had had a total abdominal hysterectomy and bilateral salpingo-oophorectomy for what she described as an ovarian cyst. Unfortunately histology from this surgery is not available, but in retrospect this may have been a granulosa cell tumour.

Following her more recent surgery an ultrasound scan revealed a residual left pelvic mass and she proceeded to receive six courses of Cisplatinum in a dose of 50 mg $m^{-2}$ combined with Chlorambucil 10 mg daily for 7 days. She had achieved complete remission on ultrasound after six courses of treatment and proceeded to receive eight courses of treatment. Three years after completing chemotherapy she was found clinically to have a mass at the vaginal vault and on CT scan a 12 × 8 cm pelvic mass was found. She was commenced on Tamoxifen, but after 4 months of treatment was shown to have progressed on CT scan with increased size of the pelvic mass associated with ascites and obstruction of the left kidney. She had an unsuccessful attempt at debulking laparotomy and was commenced on Megestrol Acetate in a dose of 160 mg daily. After 5 months of treatment she was found to have had a marked response on CT scan particularly on the left side of the tumour with resolution of her ascites.

These cases again demonstrate that progestagens can induce tumour regression in advanced cases of granulosa cell tumours and support the view expressed by Malik and Slevin that this drug could be considered as first line therapy for patients who have relapsed and in whom surgery is inappropriate.

R. Isaacs
G. Forgeson
S. Allan
Radiotherapy and Medical Oncology Department,
Palmerston North Hospital,
Ruahine Street,
Palmerstone North,
New Zealand.

**Reference**

MALIK, S.T.A. & SLEVIN, M.L. (1991). Medroxyprogesterone acetate (MPA) in advanced granulosa cell tumours of the ovary – a new therapeutic approach? *Br. J. Cancer*, **63**, 410.